# Theories on the Pathogenesis of Endometriosis

**DOI:** 10.1155/2014/179515

**Published:** 2014-02-12

**Authors:** Samer Sourial, Nicola Tempest, Dharani K. Hapangama

**Affiliations:** ^1^Department of Women's and Children's Health, Institute of Translational Medicine, University of Liverpool, Liverpool L69 3BX, UK; ^2^Centre for Women's Health Research, Liverpool Women's Hospital NHS Foundation Trust, Liverpool L8 7SS, UK

## Abstract

Endometriosis is a common, chronic inflammatory disease defined by the presence of extrauterine endometrial tissue. The aetiology of endometriosis is complex and multifactorial, where several not fully confirmed theories describe its pathogenesis. This review examines existing theories on the initiation and propagation of different types of endometriotic lesions, as well as critically appraises the myriad of biologically relevant evidence that support or oppose each of the proposed theories. The current literature suggests that stem cells, dysfunctional immune response, genetic predisposition, and aberrant peritoneal environment may all be involved in the establishment and propagation of endometriotic lesions. An orchestrated scientific and clinical effort is needed to consider all factors involved in the pathogenesis of this multifaceted disease and to propose novel therapeutic targets to reach effective treatments for this distressing condition.

## 1. Introduction

Endometriosis is a chronic, benign, oestrogen-dependent inflammatory disease affecting approximately 10% of reproductive age women and 35–50% of women with pelvic pain and infertility [1]. It can be a debilitating disease with symptoms of dysmenorrhoea, dyspareunia, and chronic pelvic pain [2].

The definition of endometriosis is histological and it requires the identification of the presence of endometrial gland and stroma-like tissue outside (ectopic) the uterus. These ectopic lesions are commonly located on the pelvic organs and peritoneum [3]. Occasionally, ectopic endometriotic lesions can be found in other parts of the body such as kidney, bladder, lungs, and even in the brain [4]. The clinical presentation of endometriosis is varied and conclusive diagnosis requires laparoscopy [5]. There has been efforts to standardize the surgical staging of endometriosis and histopathological changes with updated modified American Fertility Society scoring [6]. However this objective surgical staging does not necessarily correlate with the clinical symptoms [7]. Furthermore, there is a severe lack of knowledge on the natural progression of the disease in women since the severity measurement will require repeated invasive surgery. There are reports of endometriosis associated with spontaneous regression, no progression [8], and progressing to ovarian carcinomas [9, 10]. At the present time no methods exist to predict future prognosis of the disease stage from initial surgical diagnosis. Endometriosis has estimated annual costs of US $12 419 per woman (approximately €9579), comprising one-third of the direct health care costs with two-thirds attributed to loss of productivity [11]. For obvious and above mentioned reasons, despite being the causal basis for over 30% of new referrals to gynaecology clinics (local data), the management of endometriosis remains difficult.

Currently, there is no curative treatment for endometriosis and clinical management of symptoms such as pain is through medical and/or surgical measures. Medical management follows the basic principle of reducing inflammation, suppressing ovarian cycles and inhibiting the effect of oestrogen. Surgical management attempts to either remove only the identified endometriotic lesions or complete excision of pelvic organs [1]. Controversies exist regarding the best method of treatment; for example, some authors have suggested that surgical excision promotes disease recurrence whilst others consider surgical excision as a way to reduce the risk of progression to severe disease or future ovarian cancer [10, 12]. Neither medical nor surgical options provide long term or universally acceptable relief for patients. Improving our current knowledge on the pathogenesis of endometriosis therefore helps the clinical and basic science researchers to identify novel more suitable targets for formulating more effective therapeutic and diagnostic means.

Many theories have been proposed to explain the pathogenesis of endometriosis and to date they all remain to be conclusively confirmed. In this review, the predisposing factors in developing endometriosis, as well as the interplay between the pathological mechanisms involved in the initiation and propagation of different endometriotic lesions, will be discussed.

## 2. Methods

### 2.1. Search Strategy and Selection Criteria

We initially searched “Pubmed” for relevant literature using the terms “endometriosis” and “pathogenesis” or “classification” for studies published from 2000 to 2013 and identified 872 manuscripts. Although those papers provided the basis for this review, for detailed understanding of the topic we extended our search to much older yet frequently referred articles. Studies that were deemed suitable by the authors included those that examined the pathophysiology of human endometriosis: from in vitro basic science (molecular, genetical and functional) studies, studies employing animal (rodent/primate) models, gene expression, and epidemiological studies.

## 3. Results 

### 3.1. Classification of Endometriosis

Interrogation of pathogenesis of endometriosis highlights the current drawbacks associated with the classification of this disease. The revised American fertility society classifies endometriosis according to multiple criteria including histopathological as well as anatomical features, distinguishing superficial endometriosis from deep lesions of the peritoneum and ovaries [13]. Deep endometriosis is defined arbitrarily as adenomyosis externa, infiltrating the peritoneum by >5 mm [14]. It is noteworthy that the current classification system is limited by observer error as well as reproducibility and this may explain the poor correlation between extent of the disease and its clinical presentation [7]. Furthermore, histological information of endometriosis is limited by the technical efficiency in endometriotic biopsy sampling and processing, particularly when the lesions are located close to organs such as ureters, bowel and bladder [5]. A separate classification system (ENZIAN score) has been recently introduced for deep infiltrating endometriosis. It is a helpful aid in describing this type of endometriosis but it needs further refinement [15]. Clinical differences between superficial and deep endometriosis have been described, where severe pain is associated with >95% of deep endometriosis as compared with superficial endometriosis [16]. Progression of superficial endometriosis has been compared to that of a benign tumour, whereas the recurrence and progression of deep endometriosis has been reported to be rare [8, 17]. Superficial and deep endometriosis has been categorised by some authors as two different diseases with different pathogeneses whereas others regard them as different manifestations of the same disease [7]. Naturally this lack of consensus with disease classification creates another ambiguity around much of the available literature on pathogenesis.

### 3.2. Retrograde Menstruation

Retrograde menstruation theory is the oldest principle explaining the aetiology of endometriosis. This theory proposes that endometriosis occurs due to the retrograde flow of sloughed endometrial cells/debris via the fallopian tubes into the pelvic cavity during menstruation [18]. However, retrograde menstruation occurs in 76%–90% of women with patent fallopian tubes and not all of these women have endometriosis [19]. The larger volume of retrograde menstrual fluid found in the pelvises of patients with endometriosis as compared with healthy women may increase the risk of endometriotic lesions implantation [20]. In non-human primate models, it is possible to induce endometriosis by inoculating autologous menstrual products simulating retrograde menstruation in the peritoneal cavity of baboons and macaques [12]. With a single inoculation of menstrual endometrial tissue directly in to the pelvic cavity, up to 46% of the animals have shown development of endometriotic lesions in the pelvic cavity [21], whereas 100% of animals developed peritoneal endometriotic lesions after two consecutive cycles of inoculations of curetted menstrual endometrium. These lesions were histologically and clinically similar to human ectopic endometriotic lesions [22]. Furthermore, in a recent study deep nodular endometriosis was generated by ectopic implantation of full thickness endometrium including the basalis layer, highlighting the involvement of the endometrial basalis layer in development of ectopic lesions [23]. However, only the well-differentiated cells from the superficial functionalis layer are shed normally with the menstrual flow, the deep endometrial basalis layer remains intact throughout the woman's life. The regeneration of endometrial functionalis after menstrual shedding is thought to originate from this basalis [24]. Therefore by placing this basalis tissue with the ability to generate endometrial functional layer in the pelvis, the non-human primate models may not completely mimic the events of spontaneous retrograde menstruation. Further evidence to support Sampson's theory come from the observation that factors obstructing menstruation, such as congenital abnormalities including imperforate hymen and iatrogenic cervical stenosis, increase retrograde menstruation and the risk of developing of endometriosis [3]. Increased retrograde menstruation through experimentally induced cervical stenosis also caused endometriosis in non-human primate models [21]. The location of superficial endometriotic lesions in the posterior aspect and left side of the pelvis may be due to the effects of gravity on regurgitated menstrual product and the anatomical position of the sigmoid colon [25]. However, this theory has been disputed in the past since it cannot explain the occurrence of endometriosis in pre-pubertal girls, newborns, or males. Neonatal uterine bleeding, occurs in the immediate postnatal period in most girls following the withdrawal of (maternal) ovarian hormones, similar to menstrual bleeding and retrograde flow of this uterine bleeding has been proposed as the reason for prepubertal endometriosis [26].

### 3.3. Metaplasia

Other theories have proposed that endometriosis originates from extrauterine cells that abnormally transdifferentiate or transform into endometrial cells. The Coelomic metaplasia theory postulates that endometriosis originates from the metaplasia of specialised cells that are present in the mesothelial lining of the visceral and abdominal peritoneum [27]. Hormonal or immunological factors are thought to stimulate the transformation of normal peritoneal tissue/cells into endometrium-like tissue [3]. The coelomic metaplasia theory may explain the occurrence of endometriosis in prepubertal girls [28]. However, the usual driving force for endometrial growth, oestrogen, is not present in the pre-pubertal girls and therefore this condition may be different from endometriosis that is found in women of reproductive age. Ectopic endometrial tissue has also been detected in female foetuses and it has been suggested that endometriosis may be the result of defective embryogenesis. According to this theory, residual embryonic cells of the Wolffian or Mullerian ducts persist and develop into endometriotic lesions that respond to oestrogen [3]. Furthermore, recent theories that are put forward suggest coelomic metaplasia to be the origin of adolescent variant of severe and progressive form of endometriosis [29]. However, this theory is imperfect due to endometriotic lesions being found in areas outside of the course of Mullerian duct. Others have also proposed that endogenous biochemical or immunological factors induce resident undifferentiated cells to differentiate into endometrial-like tissue in ectopic sites resulting in endometriosis [30]. This suggestion is supported by the studies describing hormone-dependent transformation of peritoneal cells into Mullerian-type cells [31].

### 3.4. Hormones

Steroid hormones should play a central role in the aetiology of endometriosis since it is a disease of women in reproductive age and not usually seen in postmenopausal women who are not on hormonal treatment [32]. Similar to the eutopic endometrium, the growth of ectopic lesions are thought to be regulated by ovarian steroid hormones. Oestrogen is the driving force of endometrial proliferation and ectopic lesions may have an increased responsiveness to oestrogen, thus enhancing the development of endometriosis [31]. Environmental toxins, such as dioxin, are implicated in the aetiology of endometriosis, which may mimic oestrogen via interacting with oestrogen receptors [32]. Furthermore, there may be a higher bioavailability of oestradiol in endometriotic tissue due to the local aromatisation of circulating androgens to oestradiol by endometriotic stromal cells and also there may be reduced conversion of oestradiol to the less potent oestrone due to the ectopic endometriotic tissue expressing decreased 17*β*-hydroxysteroid enzymes [3]. These factors may explain the proliferative promoting phenotype described in the ectopic endometriotic tissue [31]. Progesterone generally counteracts the proliferation promoting action of oestrogen in the eutopic healthy endometrium. Many authors believe that endometriosis is associated with resistance of the endometrium to progesterone which plays a pivotal role in the pathogenesis [33, 34]. The harnessing of the oestrogen-driven mitotic/proliferative action on the endometrium by progesterone during the secretory phase of the cycle does not occur in the endometriotic lesions and sustained proliferative activity is seen in the eutopic endometrium of women with endometriosis in the secretory phase [35, 36]. The progesterone resistance may be due to the endometriotic lesion having a lower expression of progesterone receptors or as a result of a functional abnormality of the existing progesterone receptors [37].

### 3.5. Oxidative Stress and Inflammation

Increased oxidation of lipoproteins has been associated with the pathogenesis of endometriosis, where reactive oxygen species (ROS) cause lipid peroxidation that leads to DNA damage in endometrial cells [38]. The presence of water and electrolytes in the increased peritoneal fluid volume in patients with endometriosis harbours the source of ROS [39]. These patients also have iron overload in their peritoneal cavities from the breakdown of haemoglobin, which in turn causes redox reactions [40]. The release of the proinflammatory heam products and the oxidative stress signals generated from the ROS cause inflammation which leads to the recruitment of lymphocytes and activated macrophages producing cytokines that induce oxidizing of enzymes and promotes endothelial growth [30]. The excess production of ROS is also accompanied by a decreased level of antioxidants that usually eliminates these molecules [38, 41]. Resulting accumulation of ROS may contribute to the propagation and maintenance of endometriosis and associated symptoms.

### 3.6. Immune Dysfunction

The observation that autoimmune diseases to be more common in women with endometriosis support the possibility that pathogenesis of endometriosis may involve a defective immune response in these patients [42]. Women with endometriosis have a higher concentration of activated macrophages, decreased cellular immunity, and a repressed NK cell function [43, 44]. The regurgitation of endometrial cells into the peritoneum triggers an inflammatory response, recruiting activated macrophages and leukocytes locally [45]. This inflammatory response may cause a defective “immune-surveillance” that prevents elimination of the menstrual debris and promotes the implantation and growth of endometrial cells in the ectopic sites [46]. Furthermore, there are suggestions that during the evolutionary process the peritoneal immune clearance that occurs in non-human primates has been lost in humans, and this may contribute to the persistence of the menstrual debris in the pelvic cavity and subsequent development of endometriosis in women [23]. The survival and resistance to immune-cell-mediated lyses of endometriotic cells are ensured by masking these ectopic cells to the immune system, where, for example, ectopic endometrial cells modulate the expression of HLA class I molecules [43, 47]. Both immune and endometrial cells secrete cytokines and growth factors, which induce cell proliferation and angiogenesis; thereby promoting implantation and growth of ectopic lesions [48]. Possibly as a consequence, women with endometriosis have higher expression of cytokines and vascular endothelial growth factors in their peritoneal fluid, which promote proliferation of endometrial cells and angiogenesis [49, 50].

### 3.7. Apoptosis Suppression and Alteration of Endometrial Cell Fate

Alteration of the endometrial cell fate to favour antiapoptotic and proproliferative phenotype is paramount for the survival of the endometrial cells in the peritoneal cavity to initiate ectopic deposits and for the maintenance of the established lesions [51]. By examining matched eutopic endometrium and ectopic lesions from women with endometriosis and in baboon with induced disease, we have recently shown that telomerase enzyme may play a central role in this altered endometrial cell phenotype [36, 51].

There is plethora of evidence suggesting an upregulation of antiapoptotic and prosurvival genes and reciprocal downregulation of the genes regulating the apoptosis pathway in ectopic endometrial cells [52]. In addition to the decreased scavenger activity, the endometrium in patients with endometriosis expresses higher levels of antiapoptotic factors [53]. The inhibition of the apoptosis of endometrial cells may also be mediated by the transcriptional activation of genes that normally promotes inflammation, angiogenesis, and cell proliferation [54].

### 3.8. Genetics

A genetic basis for the development of endometriosis is suggested by the reports of familial aggregation, the high risk of endometriosis in those with an affected first-degree relative [55], and the observations of concordance of endometriosis in twins [56]. A great number of studies have related genetic polymorphisms as a factor that contributes to the development of endometriosis. Endometriosis has a polygenic mode of inheritance that is likely to involve multiple loci and some chromosomal regions were reported to be associated with the corresponding endometriosis phenotype [57]. Inherited as well as acquired genetic factors may predispose women to the attachment of ectopic endometrial cells to the peritoneal epithelium and the evasion of these lesions from immune clearance [3]. Differences in genes and protein expression between patients with and without endometriosis have been reported [58]. Genes that have been implicated in the pathogenesis of endometriosis include those encoding detoxification enzymes, polymorphism in oestrogen receptor, and genes involved in the innate immune system [31]. Genetic predisposition can increase the frequency of cellular damage. Genetic mutations that cause cell damage are implemented in the progression of endometriosis, since women with endometriosis show altered endometrial cell behaviour, favouring extrauterine adhesion and growth [16]. Over the past decade several authors have employed gene arrays to identify endometriosis related genes. Using laser capture microdissection and high throughput and high resolution comparative genomic hybridization (CGH) arrays, considerable genomic alterations in both eutopic and ectopic endometria of women with endometriosis have been identified [59, 60]. Recent genomewide association studies have also identified new loci to endometriosis [57]. Collectively this data suggests that different types of endometriosis may be associated with altering different gene clusters that regulate specific cellular functional aberrations.

### 3.9. Stem Cells

The monthly regeneration of the endometrium after menstrual shedding, reepithelialisation of the endometrium after parturition or surgical curettage, supports the existence of a stem cell pool [61]. Since the basalis layer of the endometrium is not shed with the monthly menstrual shedding of the functional layer, the stem cells are thought to reside in the basalis layer of the endometrium [62]. Recently, clonogenic cells, which are thought to represent the stem cell population in the human endometrium have been identified and proposed to be involved in the formation of ectopic endometrial lesions [63].

Stem cells are undifferentiated cells, characterized by their ability to self-renew and differentiate into one or several types of specialized cells [64]. Differentiation is defined as a change in cell phenotype secondary to alteration in the cell's gene expression, enabling the cell to have a specific function [28]. Endometrial self-generation may occur through stem cells in specific niches of the endometrium [65]. The undifferentiated endometrial stem cells may be less responsive to ovarian steroids than the terminally differentiated progeny due to lack of expression of hormone receptor [66]. In addition to the resident endometrial stem cells, incorporation of circulating bone marrow-derived stem cells may contribute to the cyclic regeneration of the endometrium [67].

The involvement of stem cells in the formation of endometriotic deposits could be as a result of abnormal translocation of normal endometrial basalis via retrograde menstruation [68]. Brosens et al. postulated that the uterine bleeding in neonatal girls contains a high amount of endometrial progenitor cells [29]. Some of these cells may deposit and survive in the peritoneal cavity after retrograde flow and may reactivate in the adolescents in response to ovarian hormones [29]. However, there is no current data on the amount of endometrial stem/progenitor cells in neonatal period when compared to the adult endometrium. Furthermore, since even the aging postmenopausal endometrium seem to have adequate amount of progenitor cells to generate a competent normal functionalis with the essential hormonal stimulation, it seems unlikely that there are significant differences in the progenitor activity between the premenopausal and postmenopausal endometrium. Leyendecker et al. [69] proposed that women with endometriosis abnormally shed the endometrial basalis tissue, which initiate endometriotic deposits after retrograde menstruation. The observation in the baboon model of endometriosis induction, where placement of the stem cell rich endometrial basalis in the pelvic cavity resulting in 100% induction of endometriosis in all animals, may further support Leyendeckers theory. If the basalis contains the stem/progenitor cells, they are likely to survive and initiate endometriotic deposits in the pelvis than the differentiated endometrial cells from the functionalis. Due to their natural ability to regenerate, these stem cells may give rise to new endometriotic deposits. The fact that women with endometriosis possibly shed significantly more of the stem-cell rich basalis layer as compared to healthy women [69], together with the similarity observed between ectopic lesions and the basalis layer [24], may support the possibility of retrograde menstruation providing an access for the endometrial stem cells to extrauterine structures [63, 69]. Alternatively, these stem cells may be transported via the lymphatic or vascular pathways to ectopic sites [70]. The fact that some of the endometrial stem cells have bone marrow origin further supports the haematogenous dissemination theory of these cells [71]. Recent studies have further suggested that mobile stem cells may be involved in endometriosis progression, where cells derived from ectopic lesions in induced endometriosis migrated to the eutopic endometrium [71]. However, since stem cells are normally expected to differentiate into mature cells in concordance with the environmental niche, the supposedly multipotential endometrial stem cells in the peritoneal cavity should differentiate in to peritoneal-type cells. It is possible that the deposition of endometrial tissue fragments containing both endometrial stem cells and their niche cells in the peritoneal cavity promote regeneration of endometrium-like tissue, due to the signal received by the stem cells from the surrounding endometrial niche cells. On the other hand, the relocation of an aberrant or committed stem cell from the endometrium to an ectopic site may also generate endometrium-like lesions. Endometrial tissue produces several chemokines and angiogenic cytokines; therefore, neovascularisation in the ectopic sites can presumably follow, thus ensuring the establishment of these lesions [72].

A further possibility of stem cell involvement in endometriosis is the transdifferentiation of the peritoneal, haematopoietic, or ovarian stem cells into endometrium like tissue. Peritoneal cavity connects directly with the uterine cavity and there is a free flow of the cytokine/chemokine rich fluid between the two environments. This direct connection may regulate the endometrium-like differentiation of resident stem cell population in the peritoneal cavity. Although possible, the reasons for such specific differentiation of the peritoneal stem cells in to endometrium-like tissue in only up to 10% of the female population remain unexplained.

## 4. Discussion

**Figure 1:**
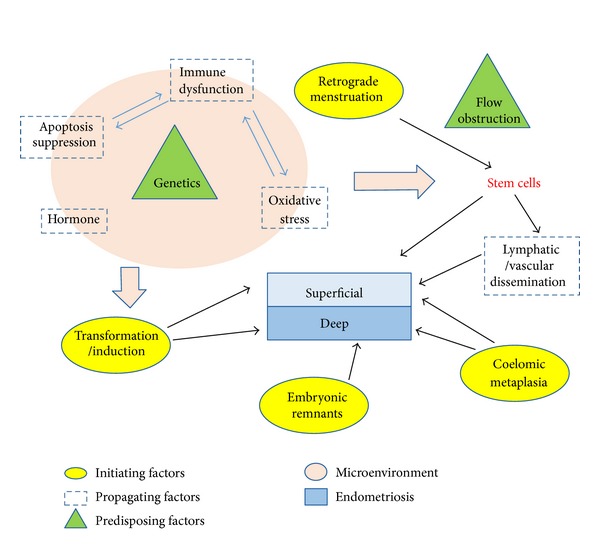
Summary of the proposed interplay between the different factors reported in the pathogenesis of superficial versus deep endometriosis. The different initiating, propagating, and predisposing factors are indicated through different shapes, respectively. The arrows indicate the interplay between the different factors. As indicated by the bold pink arrows, some of the labelled propagating factors create a microenvironment that impacts the differentiation of stem cells and/or the transdifferentiation of peritoneal cells into endometrial cells.

The different theories implicated in the pathogenesis of endometriosis indicate that the aetiology of endometriosis is complex and multifactorial, involving hormonal, genetic, immune, and environmental components.  Table 1 summarizes the role of each theory in the pathogenesis of endometriosis. While retrograde menstruation may be one of the initiating steps in the pathogenesis of superficial endometriosis, genetic and microenvironmental factors that prevent clearance of ectopic lesions and allows remodelling of peritoneum are essential for the propagation of endometriotic lesions [73, 74]. Pathogenesis of endometriosis is propagated by an altered peritoneal fluid composition as a result of genetic, hormonal, and environmental factors [75, 76].  Figure 1 depicts the interplay between the different factors that may be involved in the pathogenesis of endometriosis.

Differences are present in the pathogenesis of deep versus superficial endometriosis. Retrograde menstruation may not explain the pathogenesis of deep endometriosis, where no deep endometrial lesions could be induced in animal models through peritoneal instillation after endocervical removal of menstrual endometrium [22]. However, deep nodular lesions, which is not usually shed at menstruation, could be readily induced with the transplantation of endometrial basalis tissue in a baboon model [23]. Other theories such as the coelomic metaplasia, induction of cellular transformation into endometrial cells, and the embryonic remnant theory may better explain the aetiology behind deep endometriosis.

## 5. Conclusion

Ectopically placed stem cells that are of endometrial or haematopoietic origin or abnormal endometrial differentiation of a resident tissue stem cell may be the first step in the establishment of an ectopic endometrial lesion. The subsequent proliferation and propagation of such lesions may also be dependent on mobile, endometrial progenitor-type cells in these ectopic lesions that are involved in initiating further lesion and also in maintaining the disease. A dysfunctional immune clearance and a genetic predisposition that allow these ectopic lesions to grow in an aberrant microenvironment may also contribute to the development of the disease. The current therapeutic regimens for endometriosis are usually based on manipulating the ovarian steroid hormones that may preferentially target terminally-differentiated ectopic endometriotic cells which would normally die off via apoptosis, while the stem cells that propagate the disease may not be affected. Improving our understanding of the pathogenesis of endometriosis will direct further future work on more appropriate therapeutic targets that can provide the much needed curative and universally acceptable treatments for endometriosis.

## Figures and Tables

**Table 1:** Role of the different theories in the pathogenesis of endometriosis.

Theory	Mechanism
Retrograde menstruation	Flow of endometrial content into pelvis, allowing implantation of endometrial lesions

Metaplasia	Transformation of peritoneal tissue/cells into endometrial tissue through hormonal and/or immunological factors

Hormones	Oestrogen-driven proliferation of endometrial lesions. Resistance to progesterone-mediated control of endometrial proliferation

Oxidative stress and inflammation	Recruitment of immune cells and their production of cytokines that promote endometrial growth

Immune dysfunction	Prevention of eliminating menstrual debris and promotion of implantation and growth of endometrial lesions

Apoptosis suppression	Promoting survival of endometrial cells and downregulation of apoptotic pathways

Genetic	Alteration of cellular function that increases attachment of endometrial cells and evasion of these cells from immune clearance

Stem cells	Initiation of endometriotic deposits by undifferentiated cells with natural ability to regenerate
